# Immune Responses and Replication of Rescued Torque Teno Virus (TTSuV1) in Mice

**DOI:** 10.3390/v17081105

**Published:** 2025-08-12

**Authors:** Md-Tariqul Islam, Brett Webb, Sheela Ramamoorthy

**Affiliations:** 1 Department of Microbiological Sciences, North Dakota State University, Fargo, ND 58102, USA; md-tariqul.islam1@ndsu.edu; 2 Department of Microbiology and Immunology, Faculty of Veterinary, Animal, and Biomedical Sciences, Sylhet Agricultural University, Sylhet 3100, Bangladesh; 3 Veterinary Diagnostic Laboratory, North Dakota State University, Fargo, ND 58102, USA; brett.webb@ndsu.edu

**Keywords:** torque teno virus, mouse, model, infection, antibody, ELISA, histopathology, lymphocytes

## Abstract

Although Torque Teno Viruses (TTVs) were initially considered to be ubiquitous members of the mammalian virome, the finding that swine TTVs (TTSuV) can act as primary pathogens elevates the possible status of swine TTVs (TTSuVs) to an emerging swine pathogen. Since their discovery, the molecular mechanisms of TTV–host interactions remain largely unknown as robust in vitro culture systems and in vivo animal models have not been available. This study was undertaken to address some of these long-standing gaps. Recombinant TTSuV1 rescued from an infectious clone was used to infect C57BL/J6 mice. Infected mice seroconverted within 15 days post-infection and mounted virus neutralizing antibody responses. Viral DNA was detected in blood and lung tissue for the duration of the study. TTSuV1 isolated from the lung tissue of infected mice productively and serially infected PK-15 cells in vitro, indicating that the treatment produced viable, replicative viral particles in the host. TTSuV1 antigen was also detected by flow cytometry in lymphocytes, including the T and B lymphocyte subsets. Infected mice exhibited mild splenic hyperplasia and lymphopenia. The ability to respond to mitogenic stimuli was highly diminished in infected mice and a striking lack of virus-specific recall responses was observed for the 30-day duration of the study. Therefore, this study is the first to provide experimental evidence that recombinant TTSuV1 rescued from an infectious clone is infective and induces immune responses in laboratory mice. This model provides a critical tool for advancing research on TTV immunopathogenesis.

## 1. Introduction

Torque teno viruses (TTVs) are members of the family Anelloviridae and a central component of the mammalian virome. They are small DNA viruses that infect humans, as well as wild and domestic animals, globally. TTV infections occur early in life and persist chronically in adulthood [1,2,3,4]. In humans, TTVs are epidemiologically linked extensively to respiratory infections, autoimmune diseases, neurological conditions, cancers, and hepatitis [2,5,6]. Recently, TTVs have been recognized as an “immuno-meter” for transplant patients as viral loads in blood are strongly correlated with immune suppression, and TTV loads in transplant patients can be used to predict rejections [7,8,9,10].

Swine TTVs (TTSuVs) are considered emerging pathogens [11] due to substantial experimental and field evidence for their role as primary or supporting coinfecting agents [4]. Like human TTVs, swine TTVs (TTSuVs) are widely prevalent in both healthy and sick production pigs. However, the rate of TTSuV infection increases dramatically in morbid pigs, suggesting that TTSuVs play a role in worsening clinical outcomes in coinfections with other agents [12]. In fact, experimental infection of gnotobiotic pigs with TTSuV1 resulted in renal and hepatic pathology, thus providing the first experimental evidence for TTVs possible role as a primary pathogen [13]. Coinfection of gnotobiotic TTSuV1 with other pathogenic porcine viruses such as porcine circovirus type 2 (PCV2) and porcine reproductive and respiratory disease syndrome virus (PRRSV) resulted in worsening of pathological lesions and clinical signs of the pathogenic virus [14,15]. Providing evidence for the potentially zoonotic nature of TTSuVs, we previously reported that 68% of human sera tested were positive for TTSuV1 DNA, and that 40% of those sera had TTSuV1-specific antibodies [16]. TTSuV1 is also able to replicate in human PBMC’s and suppress lymphocyte proliferation responses, suggesting a laxity in the species-specificity of TTVs [16]. Moreover, TTVs are also common contaminants of the environment, water sources [17], pork products, and biologicals like vaccines [18,19,20,21]. Therefore, gaining a better understanding of the role of TTVs in promoting disease becomes important.

Comprehensive studies into the molecular mechanisms of host–TTV interactions have been traditionally limited by the lack of reliable in vitro cell culture systems and suitable animal models for in vivo studies [22]. We recently reported that high titers of TTSuV1 could be produced in cell culture by rescuing of recombinant TTSuV1 from an infectious clone with supplementation of overexpressed replicase protein derived from another related small DNA virus called porcine circovirus type 1 (PCV1) [23], thus addressing the gap associated with culturing TTV to high titers in vitro. While it has been previously shown that the direct injection of cloned TTSuV DNA into the lymph nodes of gnotobiotic pigs resulted in seroconversion and viral replication [24], all previously published studies on experimental infections with TTSuVs were carried out in caesarian-derived, colostrum-deprived (CD-CD) piglets [13,14,15,24]. The extremely laborious and expensive nature of CD-CD piglet model, and lack of a comprehensive immune tool kit for pigs are major limiting factors in using pigs for TTV research. Thus, the primary goal of this study was to develop a more reproducible and convenient mouse model for experimental TTSuV1 infection and establish baselines for measuring viral replication and immune responses. Here, we report the first successful development of a murine model of TTSuV1 infection, enabling controlled and mechanistic investigations into TTV-induced immunopathogenesis and coinfection dynamics.

## 2. Materials and Methods

### 2.1. Viruses and Cells

The preparation of the TTSuV1 infectious clone and rescue of recombinant TTSuV1 was as previously described [23,25]. Briefly, the TTSuV1 genome (GenBank: KT037083.1) was amplified from the bone marrow of a pig with clinical signs of the porcine respiratory disease complex [16] and cloned into the TA cloning vector (Thermo Fisher Scientific, Waltham, MA, USA). An amino acid sequence encoding the V5 tag (GKPIPNPLLGLDST) was inserted at the 3′ end of the ORF1 gene to serve as a serological and genetic marker. The TTSuV1 genome was dimerized to avoid excision and recircularization of the viral genome for rescue [23]. The dimerized genome was transfected into porcine kidney cells (PK15N, 005-TDV, National Veterinary Services Laboratory, Ames, IA, USA) to rescue recombinant TTSuV1 as described before [23,25,26]. As TTSuV1 is non-cytolytic, the rescued virus was visualized by an immunofluorescence assay (IFA) using a TTSuV1-specific antibody as described before [16,23,25]. The virus culture was titrated by the TCID_50_ method and stored at −80 °C until further use.

### 2.2. Infection of Mice with TTSuV1

A total of 14, 2–3 weeks old C57BL/J6 mice (Jackson laboratory, Bar Harbor, ME, USA) were divided into two groups: (A) Uninfected (PBS) (N = 4) and (B) TTSuV1 (N = 10). Mice from the TTSuV1 group were infected with 10^4^ TCID_50_/mL recombinant TTSuV1, 50 µL intranasally and 250 µL intra-muscularly on day 1 post-infection (DPI 1). Mice in the negative control group were administered the same volume of PBS by the same routes. The mice were observed daily for clinical signs or post-infection complications. Whole blood was collected on day DPI 0, 7, 15, 22, and 30 post-inoculation (DPI) for qPCR and flow cytometry, while serum was collected on DPI 0,15, and 30 to assess antibody responses. On DPI 15, half of the mice from each group were euthanized and major organs were collected for histopathological evaluation. Splenic lymphocytes were collected for lymphocyte proliferation assays. The remaining mice were euthanized on DPI 30 and samples collected as described above. All animal experimentation was carried out in compliance with North Dakota State University’s institutional IACUC policies and procedures.

### 2.3. Daily Post-Infection Observations

The mice were observed daily for clinical signs like general condition, liveliness, ruffled coats, huddling, ocular discharge or staining, nasal discharge, inappetence, loss of body weight, and condition. Scores for the clinical signs were assigned on a scale of 1 = Mild, 2 = Moderate, 3 = Severe. Body temperatures were measured for 3 days post-infection.

### 2.4. TTSuV1-Speciic Antibody Responses

**Figure 1:**
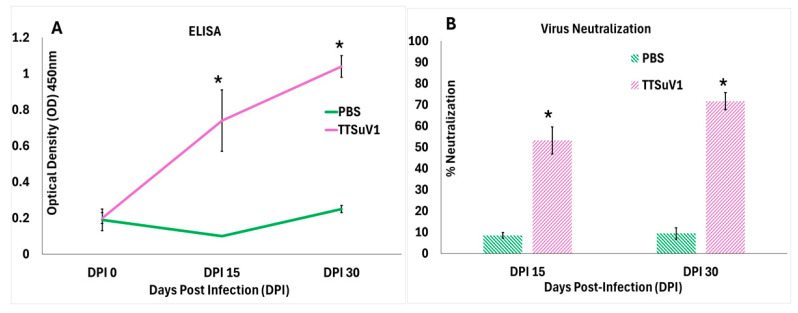
Serological responses to TTSuV1 infection in mice. (**A**) Binding antibody responses as measured by a TTSuV1-specific ELISA. *X*-axis—days post-infection (DPI), *Y*-axis—Mean optical density (OD), Green line—Uninfected mice, Pink line—TTSuV1-infected mice, * *p* ≤ 0.05 when compared to the uninfected control group, Student’s *t*-test. (**B**) Virus neutralizing antibody responses measured by a fluorescent focus inhibition assay. *X*-axis—days post-infection (DPI), *Y*-axis—mean % virus neutralization, green bar—Uninfected pigs, pink bar—TTSuV1-infected pigs, * *p* ≤ 0.05 when compared to the uninfected control group by Student’s *t*-test.

Evaluation of anti-TTSuV1 IgG responses was performed as described before [23,26]. Briefly, 96-well ELISA plates (High Bind Microplate, Corning^®^, Corning, NY, USA) were coated with 1:100,000 dilution of recombinant TTSuV1 ORF2 antigen overnight. The plates were blocked with General Block (Immunochemistry Technologies, Bloomington, MN, USA) containing 2% BSA and 2% goat serum for 2 h at 37 °C. Test serum was diluted to 1:50 and added in duplicate to the coated plates and incubated for 2 h at 37 °C. Two separate assays were conducted to obtain a total of 4 values per sample. Following incubation, goat anti-mouse IgG HRPO conjugate (1:5000) (KPL, Milford, MA, USA) was added and incubated for 45 min. Color development was achieved using TMB substrate (KPL, Milford, MA, USA). The reaction was stopped with 1 M HCl solution. Plates were read at 450 nm on an ELISA plate reader (Elx800 reader, BioTek Instruments, Inc., Winooski, VT, USA). Positive and negative controls were included in each assay (Figure 1A).

### 2.5. Virus Neutralization Assay

As TTSuV1 is non-cytolytic, a micro fluorescent focus neutralization (FFN) assay was used to measure functional neutralizing antibodies as previously described [27]. Briefly, the rescued TTSuV1 virus was reconstituted to 10^3^ TCID_50_/mL. Fifty to sixty fluorescent foci were produced when 100 µL of the reconstituted culture was cultured on PK15 cells in a 96-well tissue culture plate. Test sera from DPI 15 and DPI 30 were heat-inactivated at 56 °C for 10 min and diluted to 1:50 in Dulbecco’s modified eagle’s medium (DMEM, Corning Cellgro, Tewksbury, MA). Equal volumes of the 1:50 diluted serum and 10^3^ TCID_50_/mL virus culture were mixed in non-adhesive U bottom plates and incubated for 1 h at 37 °C. The virus–serum mixture was layered on 40–50% semi-confluent PK-15 monolayer cells in a 96-well culture plate and incubated for 36 h at 37 °C. Virus only and media controls were included in each assay. After the incubation, the cells were fixed with ice-cold acetone: methanol (1:1). Fixed cell sheets were stained with a 1:200 dilution TTSuV1-speciifc rabbit antibody at 37 °C for 1 h, followed by detection with FITC-labeled anti-rabbit IgG (1:100) for 45 min. Each sample was assessed in duplicate. Fluorescent foci were counted in a blinded fashion using a fluorescent microscope (Cytation 5, BioTek). The FFN titer was expressed as the percentage reduction in foci in the test samples compared to the total virus only control (Figure 1B).

### 2.6. Viral Genomic DNA Quantification

**Figure 2:**
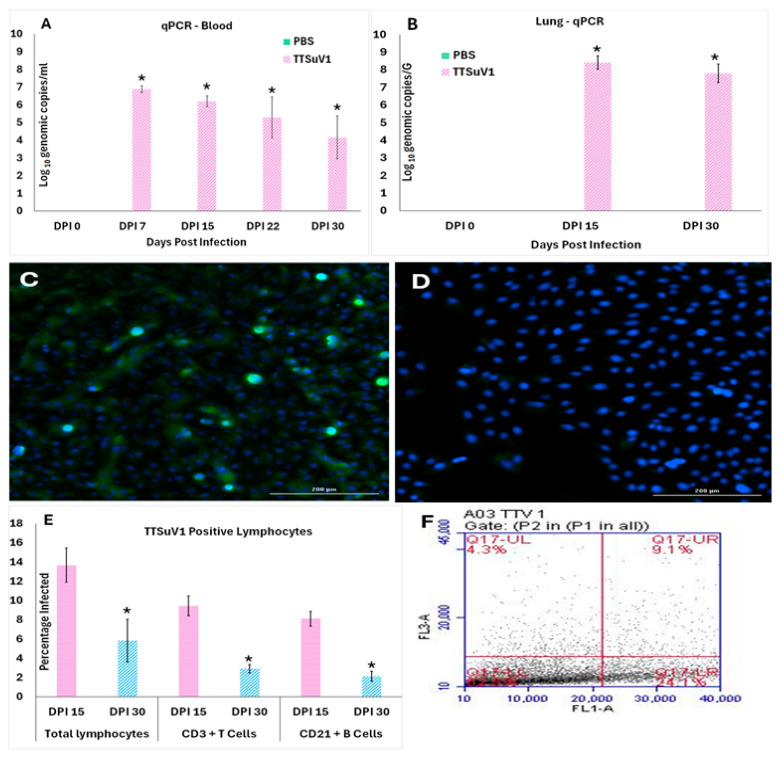
TTSuV1 replication in infected mice. 2A and B. Detection of TTSuV1 genomic DNA by a TTSuV1-specific qPCR assay. (**A**) Detection in whole blood. (**B**) Detection in lung tissue. *X*-axis—Days post-infection (DPI), *Y*-axis—Mean log_10_ TTSuV1 genomic copy numbers, green bar—PBS group, pink bar—TTSuV1 group, * *p* ≤ 0.05 when compared to the uninfected control group, Student’s *t*-test. (**C**,**D**) Virus isolation from lung tissues. Representative IFA images from passage 1 of the DPI 15 lung virus isolation showing green, fluorescent nuclei characteristic of small DNA viral replication 2C. Virus isolation from the lungs of TTSuV1-infected mice 2D. Virus isolation from the lungs of the PBS group. (**E**,**F**) Detection of TTSuV1 antigen in lymphocytes by flow cytometry. (**E**) *X*-axis—Cell types and days post-infection (DPI), *Y*-axis—Mean percentage of infected cells, Pink—DPI 15, Blue—DPI 30, * *p* ≤ 0.05 when compared to the DPI 15 values by Student’s *t*-test. (**F**) Representative image of a flow cytometry quadrant plot showing the percentage of cells which are double positive for the CD3 marker and intracellular TTSuV1-specific stain in the upper right corner, indicative of infected CD3+T cells.

A TTSuV1-specific qPCR assay was used to quantify the copy numbers of TTSuV1 genomic DNA in whole blood and lung samples. DNA was extracted from whole blood using the QIAamp Blood Minieasy Kit (Qiagen, Valencia, CA, USA) and lung tissue using the QIAamp DNA tissue Kit (Qiagen, Valencia, CA, USA), respectively, following the manufacturer’s protocols. The qPCR reaction consisted of 2 µM probe 5′FAM/CACACAACACAGCAGGAA/3IABkFQ3′, 0.4 µM of each primer (5′ TACCCGGCTTTGCTTCGACAGTG3′), and 5′GCCATAGATTTCTAGCGATCCCAATTGCG-3′) at a Tm of 57 °C using 10 µL DNA with the QuantiFast Probe PCR mix (Qiagen, Valencia, CA, USA). The thermocycling conditions included a hot start at 95 °C for 5 min, 35 cycles of denaturation at 95 °C for 15 s, annealing for 57 °C for 30 s, and extension for 72 °C for 30 s in a thermocycler (CFX96 Touch, Bio-Rad, Hercules, CA, USA). The data obtained were analyzed using the CFX manager software (Bio-Rad, Version 3.1). Logarithmic dilutions of a known quantity of plasmid DNA encoding the TTSuV1 genome were used to obtain a standard curve, which was used to convert Ct values to copy numbers. No template and plasmid positive controls were included in each assay (Figure 2A,B). The lowest detection limit was 44 genomic copies/mL.

### 2.7. Virus Isolation

To verify that replicative TTSuV1 was produced by experimental infection DPI 15 and 30 lung tissue from the PBS and TTSuV1 groups were used for virus isolation. 30–50 mg of flash frozen lung tissue samples were pooled and macerated through 0.22 μm pore size syringe filter (Millex™ MCE syringe filter-Millipore Sigma) in DMEM containing 1X penicillin–streptomycin solution. Following 3 freeze–thaw cycles, the lysates were centrifuged at 2500 rpm for 7 min. 80 μL of the supernatant was layered on 50% confluent pig kidney cells (PK-15, CCL3-ATCC, Manassas, VA, USA) and incubated for 72 hrs. The isolated TTSuV1 culture was passaged three consecutive times. TTSuV1 replication was visualized by an immunofluorescence assay carried out as described above (Figure 2C,D). A TTSuV1 virus control reconstituted to 1 × 10^3^ TCID_50_/mL titer served as a positive control while lung lysates from the PBS group served as negative controls.

### 2.8. Flow Cytometry for the Detection of TTSuV1 Antigen and Changes in Lymphocyte Counts

**Figure 3:**
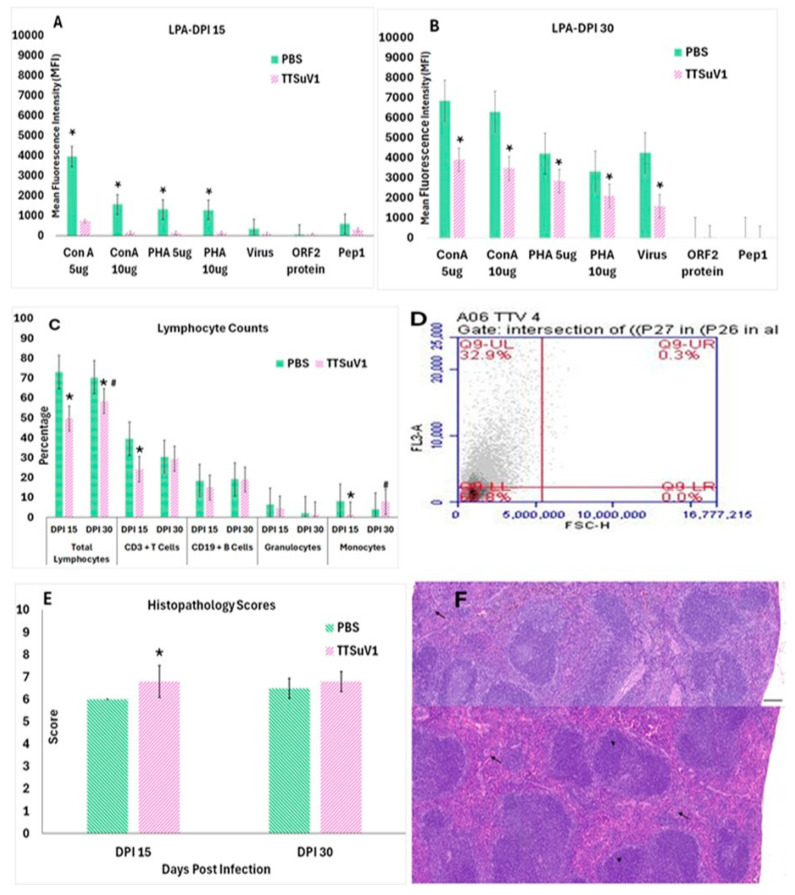
Effect of TTSuV1 infection on lymphocytes: (**A**,**B**) Lymphocyte proliferation assay. Lymphocytes from whole blood collected at DPI 15 and 30 were stimulated with either mitogens or viral antigens. *X*-axis—treatments, *Y*-axis—Mean fluorescence intensity (MFI), Green—PBS group, Pink—TTSuV1 group, * *p* ≤ 0.05 when compared to the uninfected control group, Student’s *t*-test. (**C**) Changes in lymphocyte percentages. The percentages of various PBMCs cell types in infected mice were compared to uninfected mice at DPI 15 and DPI 30 by flow cytometry. *X*-axis—Cell types and days post-infection (DPI), *Y*-axis—Mean percentage of cells, green—PBS group, pink—TTSuV1 group, * *p* ≤ 0.05 when compared to the DPI 15 values by Student’s *t*-test. # *p* ≤ 0.05 when compared to the PBS group values by Student’s *t*-test **(D**) Representative image of a flow cytometry quadrant plot from a TTSuV1-infected mouse at DPI 30, showing the percentage of cells identified as positive for the CD3 marker in the upper left corner. (**E**) Evaluation of microscopic lymphoid lesions. Spleen sections from infected and uninfected mice were scored for cellularity using the scoring system: marked hypoplasia = 1, moderate hypoplasia = 2, mild hypoplasia = 3, Normal = 4, mild hyperplasia = 5, moderate hyperplasia = 6, Marked hyperplasia = 7. Extramedullary hematopoiesis was scored as follows: Normal = 1, mild = 2, moderate = 3, Marked = 4. The mean of the cumulative score for cellularity and extramedullary hematopoiesis is represented. *X*-axis—days post-infection (DPI), *Y*-axis—Mean score, pink—TTSuV1-infected mice, green—PBS group, * *p* ≤ 0.05 when compared to the uninfected control group. (**F**) Representative H&E-stained sections of spleen depicting lymphoid hyperplasia (arrow heads) and extramedullary hematopoiesis (arrows). Top—Uninfected mouse with a score of 4 = normal, Bottom—Infected mouse with a score of 5 = mild lymphoid hyperplasia. Scale bar = 300 µm.

Whole blood collected at DPI 15 and DPI 30 was diluted as 1:1 with PBS. RBC’s were lysed with 1× BD PharmLyse™ (Cat. No. 555899, BD Bioscience, San Jose, CA, USA) for 10 min at room temperature (RT) in the dark. The remaining cells were counted by the standard trypan blue assay and reconstituted to 1 × 10^6^ cells per 100 µL reaction. The cells were blocked by Mouse BD Fc Block™ Clone 2.4G2 (BD Bioscience) at 1 µg/million cells to reduce Fc-mediated binding and then stained for surface markers. Fluorochrome-conjugated mouse CD3e Monoclonal Antibody (OKT3 PE, eBioscience™) was used as a pan T cell marker at 0.25 µg/100 µL. Anti-CD19 PerCP-Cy5.5 647 (eBioscience™) was used as a B cell marker at a concentration of 0.5 µg/100 µL. Cells were fixed with 4% PFA for 30 min at 4 °C. Intracellular staining with rabbit polyclonal anti-TTSuV1 Ab was carried out with a 1:100 dilution of the antibody, for 45 min at RT. FITC-conjugated anti-rabbit IgG (KPL, Gaithersburg, MD, USA) was used at a 1:50 dilution in 1X BD Perm/Wash™ (BD Bioscience, San Jose, CA, USA) solution for detection. Flow cytometry analysis was conducted in a high-speed cell sorter (BD Accuri™ C6 Plus Flow Cytometer, BD Bioscience, EU) and 100,000 events were counted following an appropriate gating strategy. Single-stained, rabbit IgG isotype controls and fluorescence-minus-one (FMO) controls were included in all assays. A low forward scatter (FSC) and low side scatter (SSC) gate was applied. After excluding dead cells and doublets, TTV-infected PBMC’s were identified by the fluorescent intensity in the FITC (FL1-A) channel. Individual cell types were selected by distribution in dot plots as count and fluorescent intensity for CD19-positive B cells within the PE gate (FL2-A) and CD3e-positive T cells identified within the PerCP-Cy5.5 (FL3A) gate (Figure 2F). Total lymphocyte, monocyte, and granulocyte counts were obtained by gating as described before [28] (Figure 3C,D).

### 2.9. Lymphocyte Recall Responses

Whole blood samples collected at DPI 15 and DPI 30 were diluted as 1:1 with PBS. RBCs were lysed with 1× BD PharmLyse™ (Cat. No. 555899, BD Bioscience) for 10 min at RT in the dark. PBMC’s were harvested and counted by a standard trypan blue assay and resuspended to 1 × 10^6^ cells/mL in 1X RPMI-1640 containing 10% FBS. To evaluate proliferation responses to mitogens, the PBMCs were stimulated with concanavalin-A (ConA) or phytohaemagglutinin (PHA) at a concentration of 5 or 10 µg/mL. To evaluate TTSuV1 specific recall responses, the cells were stimulated with heat-inactivated TTSuV1 virus at 1 × 10^5^ TCID_50_/mL, recombinant ORF2 protein [16,29], a peptide encoding the first 117 amino acids of TTSuV1 ORF1 designated as peptide 1, ORF1 amino acids 6 RWRRRLGRRRRRYRK 20 designated as peptide 2, and ORF1 amino acids 305 TNTDNKNYNVNEGEEK 320 designated as peptide 3, each at a concentration of 25 µg/mL. Assays were run in triplicate and included media only control wells to obtain the baseline data. The proliferative responses were measured using the Alamar Blue reagent (AbD Serotec/Bio-Rad, Raleigh, NC, USA) [30]. Plates were read after 6 hrs incubation at 37 °C at 570–600 nm wavelength. The mean fluorescence intensity (MFI) values were obtained after subtraction of the media only control values for each mouse as represented in Figure 3A,B.

### 2.10. Gross and Microscopic Pathological Evaluation

Gross changes in the major organs were evaluated during necropsy. Liver, kidney, spleen, heart, lungs, large intestine, and ileum were fixed in 10% formaldehyde for histopathological analysis. Following fixation, tissue sections were trimmed, processed, and embedded in paraffin and cut into 5 μm thick sections and stained with hematoxylin and eosin. The stained sections were evaluated in a blinded fashion by a board-certified veterinary pathologist. Splenic lymphoid hyperplasia or hypoplasia and extramedullary hematopoiesis were evaluated as described before [31,32]. The scoring system used was as follows: Marked hypoplasia = 1, moderate hypoplasia = 2, mild hypoplasia = 3, Normal = 4, mild hyperplasia = 5, moderate hyperplasia = 6, Marked hyperplasia = 7. Extramedullary hematopoiesis was scored as follows: Normal = 1, mild = 2, moderate = 3, Marked = 4 (Figure 3E,F).

### 2.11. Data Analysis

Statistical analysis was carried out using Microsoft Excel 17.0 or Minitab software 22.0. Data were assessed for normal distribution and significance levels were set at *p* < 0.05 for all tests. Quantitative data were analyzed by the parametric Student’s *t*-test. The figures and graphs include mean or consolidated adjusted values with standard deviations and statistical significance for differences between test groups.

## 3. Results

### 3.1. Mice Are Productively Infected with Recombinant TTSuV1

When TTSuV1-specific antibody responses were evaluated by an ELISA, anti-TTSuV1 IgG was detected in sera of infected mice as early as DPI 15 with an upward trend until DPI 30 when the study was terminated. Evaluation of functional virus neutralizing antibody responses by a fluorescent focus inhibition assay revealed that infected mice mounted significant levels of virus-neutralizing antibodies by DPI 15, which increased progressively. Significant binding or virus-neutralizing antibody responses were not detected in the uninfected mice (Figure 1). As expected, both the infected mice and the uninfected control mice remained clinically normal throughout the study period, with no significant differences in bodily condition or temperature scores.

To assess infectivity of recombinant TTSuV1 for C57BL/J6 mice, we quantified viral DNA in blood and lung tissue via qPCR, isolated viable virus from lung tissue, and confirmed lymphotropic infection using flow cytometry (Figure 2). The copy numbers of TTSuV1 genomic DNA were highest at DPI 7 and reduced over subsequent time points in whole blood. However, significant levels of genomic DNA continued to be detected until DPI 30 when the study was terminated (Figure 2A). Lung tissue was collected from the experimental mice at DPI 15 and DPI 30, when mice were necropsied to evaluate pathology. TTSuV1 DNA levels in lung tissue exceeded those in blood at both DPI 15 and 30. Unlike blood, no significant decline was observed over time in the lung (Figure 2B). As expected, the mice from the PBS group remained PCR-negative throughout the study.

The detection of DNA alone may not provide conclusive evidence for the production of complete and infectious TTSuV1 particles due to the experimental infection with the rescued TTSuV1. Hence, lung tissue was used to isolate and passage TTSuV1 in vitro in PK-15 cells, thereby demonstrating the recovery of viable virus from the infected mice. Bright green nuclear fluorescence typical of the replication of small DNA viruses was clearly visible in all three passages tested using a TTSuV1-specific immunofluorescence assay (IFA) (Figure 2C). Lung tissue lysates from the PBS group did not yield viable TTSuV1 and remained negative by IFA (Figure 2D). Due to limited sample volumes, virus isolation from whole blood was not feasible.

As TTVs are believed to be primarily lymphotropic [33], active infection of immune cells was evaluated by flow cytometry where the presence of TTSuV1 antigen in PBMCs was identified using a TTSuV1-specific rabbit antibody. 13.5%, 9.5%, and 8.0% of total lymphocytes, CD3+T cells, and CD19+B cells, respectively, were positive for TTSuV1 antigen at DPI 15. While the intracellular TTSuV1-specific signal was detected in all three cell types at DPI 30, the percentage of antigen positive lymphocytes significantly declined at the later time point (Figure 2E,F).

### 3.2. Infection with TTSuV1 Diminishes Lymphocyte Function

The immune effects of TTSuV1 infection were further evaluated by testing the ability of lymphocytes from infected mice to respond to mitogenic or TTSuV1-specific antigenic stimuli (Figure 3A,B), comparing differential leukocyte counts in infected versus uninfected mice by flow cytometry (Figure 3C,D) and microscopic evaluation of splenic lymphoid lesions by a board-certified pathologist (Figure 3E,F). All three methods used to assess lymphocyte function revealed that TTSuV1 infection compromises lymphocyte function significantly. Lymphocyte proliferation responses to stimulation by mitogens were severely repressed in TTSuV1-infected mice at DPI 15 and remained significantly lower than that of the mice in the PBS group for the duration of the study. Furthermore, recall responses to inactivated whole virus or selected viral antigens representing 2 major viral proteins were either completely absent or detected at very low levels, indicating that priming of the virus-specific cell mediated immune response was severely compromised in TTSuV1-infected mice at the time points of this study (Figure 3A,B).

To evaluate whether the detection of TTSuV1 antigen in the lymphocytes of infected mice resulted in a loss of immune cells, the total number of lymphocytes, CD3+T cells, CD19+B cells, monocytes, and granulocytes were enumerated by flow cytometry. The counts of all the cell types examined were lower in infected mice compared to the uninfected controls at DPI 15. Although the percentage of lymphocytes remained lower in infected mice at DPI 30, the difference between infected and uninfected mice was not statistically significant, except for the total lymphocyte count. Infection with TTSuV1 appeared to induce monocytosis by DPI 30 as the number of monocytes in infected mice was significantly higher than those of the uninfected mice at DPI 30 (Figure 3C,D). As expected, severe gross or microscopic lesions typical of highly virulent viral infections were not observed in the TTSuV1 infected mice. Histopathological evaluation of spleens revealed mild lymphoid hyperplasia and extramedullary hematopoiesis in TTSuV1-infected mice, suggesting reactive immune activation in the absence of overt viral pathology (Figure 3E,F).

## 4. Discussion

Experimental evidence supporting the role swine TTVs (TTSuVs) as potentially emerging primary pathogens and coinfecting agents is direct and compelling [11,13,15]. Similarly, numerous studies on human TTVs provide indirect evidence for the pathogenic potential of human TTV’s, particularly in the context of immuno-suppression [10] and coinfection [1,5]. For example, a recent study reports that members of Anelloviridae are significantly enriched in the ileal virome of Crohn’s disease patients. Passive transfer of the gut virome from Crohn’s patients resulted in proinflammatory cytokine production and exacerbation of intestinal inflammation in mice [34]. Therefore, improved understanding of the molecular mechanisms by which TTVs interact with their hosts has important implications for several areas of health, including the pathology of coinfections and comorbidities, immune responses to infection and vaccination, virome-mediated microbial homeostasis, blood transfusion, and organ transplantation. The difficulty associated with developing reliable cell culture methods and animal models has been a long-standing challenge for TTV researchers. This study provides the first direct evidence that our lab-based rescued recombinant TTSuV1 can establish a productive infection and modulate host immunity in a laboratory mouse model.

The availability of a pure culture of an infectious agent is critical for the accurate interpretation of in vivo study findings. However, the difficulty in obtaining high enough titers of replicative TTV likely led to the use of blood or tissue lysates from PCR-positive animals or humans as the inoculum in previously published reports on experimental TTV infections in chimpanzees or pigs. While the absence of other major pathogens was confirmed by PCR, NGS was not used to obtain a comprehensive microbial profile of the inoculum in these studies [13,14,15,35], thus making the interpretation of results difficult. In one study, the lymph nodes and muscles of pigs were directly injected with cloned TTSuV DNA. With this approach, making a distinction between antibody responses to viable and replicative virus from responses to proteins expressed from DNA, as well as distinguishing between mere detection of the injected DNA from viral DNA by qPCR [24], became a challenge. The only published work on experimental infection of TTVs in mice is a study by Isaeva et al., (27) where blood from TTV-positive humans was used to infect mice. TTV DNA could be detected by PCR in the infected mice by 20 days and in several major organ systems for the 80-day duration of the study [36]. However, the use of infected blood for experimental studies is not ideal as the inoculum is highly likely to carry other agents. By optimizing an in vitro system for the rescue of TTSuV1 from an infectious clone [23], we were able to produce pure culture of this agent for this study and demonstrate, for the first time, that the recombinant TTSuV1 successfully replicated and induced immune responses in laboratory mice.

As the frequency of TTV detection in both healthy and sick humans and animals is high [4,12,37], overt clinical signs were not expected or observed in the TTSuV1-infected mice. An inverse correlation between the level of circulating TTV antibodies and TTV viral loads and severity of disease has not been observed in field studies [11,35,38,39]. Aligned with these findings, although seroconversion and virus-neutralizing antibodies were detected by DPI 15 (Figure 1), they were insufficient to prevent ongoing viral replication and result in clearance by DPI 30. However, the decline in TTSuV1 genomic DNA copy numbers over time in blood samples but not lung tissues suggest that increasing antibody responses resulted in reducing viremia (Figure 2A), but it was not effective in limiting infection in the lung (Figure 2B). Recent studies show that TTV loads of ≥4 log copies/mL are associated with increased mortality in patients with chronic obstructive pulmonary disease (COPD). Like other single-stranded DNA viruses that cause host DNA damage during replication TTV loads ≥6 log copies/mL were associated with increased DNA damage in COPD patients [40,41]. TTVs are known to cause systemic infections as TTVs’ DNA is detected in a variety of tissues, blood, secretions, and excretions in infected hosts [42,43,44,45,46,47]. Testing of all the tissues collected during necropsy for the presence of TTSuV1 DNA was not undertaken and is a limitation of this study. The current classification of TTVs is species-specific. Endogenous rodent TTVs (RoTTVs) have been detected in voles, wood mice, and rats but not in domestic or laboratory mice [48,49]. Thus, the likelihood of the presence of an endogenous as-yet-undiscovered mouse TTV species in the study animals was very low but could not be excluded. Therefore, to ensure the identity of the detected TTSuV1 genomic DNA matched that of the viral inoculum, the probe used in the qPCR assay was specifically designed to hybridize to the genomic and antigenic V5 marker introduced in the TTSuV1-infectious clone [16], thus ensuring that the identity of the detected TTSuV1 genomic DNA matched that of the viral inoculum.

As the mere presence of viral DNA alone is not conclusive evidence that the experimental treatment resulted in productive infection, virus isolation from the lung tissue and passaging of the TTSuV1 isolates in cell culture confirmed that viable TTSuV1 was indeed generated in infected mice. Furthermore, a TTSuV1-specific antibody prepared by immunizing rabbits with a peptide localizing to the ORF1 of the infectious clone [16,23] was used to verify the identity of the isolates by IFA (Figure 2C,D). While it is known that TTV infections are systemic [36,43], a limitation of this study is that virus isolation was not carried out in blood and other tissues. As several previous studies suggest that lymphocytes are sites of predilection for TTVs [33], the ability of TTSuV1 to directly infect lymphocytes was assessed by flow cytometry, using antibodies against cell type-specific surface markers and the TTSuV1 ORF1-specific antibody for intracellular antigen detection. Consistent with other findings [50,51], TTSuV1-specific fluorescent signals were detected in total lymphocytes, CD3+T cells and CD19+B cells (Figure 2E,F), confirming lymphotropic infection, with both T and B cells harboring TTSuV1 antigen. The decline in antigen-positive cells mirrored reduced viremia by DPI 30 (Figure 2A), suggesting that viral clearance in blood begins by DPI 30 (Figure 2D,E).

Virus induced suppression of the mitogenic response is an indicator of immuno-suppression, and is well-documented in the literature for several viruses including measles, cytomegalovirus, and lymphocytic choriomeningitis [52]. The effect can be transient or long-term and is likely critical in establishing chronic infections. In the case of TTSuV1, the suppression of the mitogenic response was evident until DPI 30 (Figure 3A,B), but long-term effects were not evaluated in this study. Notably, the striking lack of recall responses to viral antigens for the duration of the study suggests that suppression of virus-specific immune responses is also a key immune evasion strategy for TTV’s, especially in early infection, and likely occurs synergistically with the generalized immuno-suppression. Indeed, a recently published study demonstrated that upregulation of the NKG2A receptor and its binding to TTV-specific peptides on TTV-infected CD8+T cells resulted in exhaustion of the CD8+T cells, as well as loss of IFN-γ and NK cell activity [53], providing compelling experimental support for our observations. However, since TTVs are a part of the virome of most healthy individuals who can adequately respond to antigenic stimuli, the effect is likely transient. The finding that TTSuV1 infecting T and B cells (Figure 2E,F) causes a reduction in cell numbers (Figure 3C,D) suggests that the immuno-suppression is at least, in part, likely to be a direct effect of infection of lymphocytes. The mild lymphoid hyperplasia noted in the spleens of TTSuV1-infected mice could be a reactive proliferation in response to the reduced immune function [54], while the extramedullary hematopoiesis observed can be related to impaired bone marrow function [55]. Indeed, high TTSuV1 viral loads in bone marrow has been previously associated with anemia in pigs [13] (Figure 3E,F). Indeed, TTV is known to be a reliable marker of immune competence in solid organ transplant patients. Recent studies show that *Torquetenominivirus* (TTMV) can also be used as a marker for immune competence [56]. While the focus of the current study is on TTSuVs, our future studies will include developing tools to study other Anelloviruses. Our attempts to optimize an immuno-histochemistry assay using the rabbit anti-TTSuV1 antibody were not successful. So, direct visualization of viral antigens in tissues was not possible. Many other viral or host mediated mechanisms such as induction of suppressor cells, expression of interfering miRNA’s, induction of immuno-suppressive cytokines or immuno-regulatory molecules such as PD1, PD-L1, CTLA-4, rapid antigenic variation, or quasi-species production, and impaired dendritic cell or antigen presentation functions, which are commonly encountered in chronic viral infections, [57] could explain the observed immuno-suppression. A more detailed characterization of the mechanisms involved will be the focus of future studies.

In conclusion, this study directly addresses the long-standing barrier posed by the lack of robust in vitro and in vivo models for TTV research. The described mouse model overcomes a critical bottleneck in TTV research by providing a reproducible, accessible system to investigate virus–host dynamics, immune suppression, and pathogenesis, while reducing cost and labor significantly when compared to the gnotobiotic pig model.

## Data Availability

Data will be made available as required.

## Abbreviations

The following abbreviations are used in this manuscript:

TTV	Torque Teno Virus
TTSuV	Torque Teno Sus Virus
PBMC	Peripheral blood mononuclear cells
TCID50	Tissue culture infective dose 50
qPCR	Quantitative polymerase chain reaction
PBS	Phosphate-buffered saline
h/hrs	Hour/hours
min	Minutes
